# A Novel Bradykinin-Related Dodecapeptide (RVALPPGFTPLR) from the Skin Secretion of the Fujian Large-Headed Frog (*Limnonectes fujianensis*) Exhibiting Unusual Structural and Functional Features

**DOI:** 10.3390/toxins6102886

**Published:** 2014-09-29

**Authors:** Daning Shi, Yu Luo, Qiang Du, Lei Wang, Mei Zhou, Jie Ma, Renjie Li, Tianbao Chen, Chris Shaw

**Affiliations:** 1 Natural Drug Discovery Group, School of Pharmacy, Queen’s University, Belfast BT9 7BL, Northern Ireland, UK; E-Mails: dshi01@qub.ac.uk (D.S.); qdu01@qub.ac.uk (Q.D.); l.wang@qub.ac.uk (L.W.); m.zhou@qub.ac.uk (M.Z.); t.chen@qub.ac.uk (T.C.); chris.shaw@qub.ac.uk (C.S.); 2 School of Medicine, Dentistry and Biomedical Sciences, Queen’s University, Belfast BT9 7BL, Northern Ireland, UK; E-Mail: yluo03@qub.ac.uk

**Keywords:** amphibian, skin secretion, molecular cloning, bradykinin, smooth muscle

## Abstract

Bradykinin-related peptides (BRPs) are significant components of the defensive skin secretions of many anuran amphibians, and these secretions represent the source of the most diverse spectrum of such peptides so far encountered in nature. Of the many families of bioactive peptides that have been identified from this source, the BRPs uniquely appear to represent homologues of counterparts that have specific distributions and receptor targets within discrete vertebrate taxa, ranging from fishes through mammals. Their broad spectra of actions, including pain and inflammation induction and smooth muscle effects, make these peptides ideal weapons in predator deterrence. Here, we describe a novel 12-mer BRP (RVALPPGFTPLR-RVAL-(L^1^, T^6^, L^8^)-bradykinin) from the skin secretion of the Fujian large-headed frog (*Limnonectes fujianensis*). The *C*-terminal 9 residues of this BRP (-LPPGFTPLR) exhibit three amino acid substitutions (L/R at Position 1, T/S at Position 6 and L/F at Position 8) when compared to canonical mammalian bradykinin (BK), but are identical to the kinin sequence present within the cloned kininogen-2 from the Chinese soft-shelled turtle (*Pelodiscus sinensis*) and differ from that encoded by kininogen-2 of the Tibetan ground tit (*Pseudopodoces humilis*) at just a single site (F/L at Position 8). These data would imply that the novel BRP is an amphibian defensive agent against predation by sympatric turtles and also that the primary structure of the avian BK, ornithokinin (RPPGFTPLR), is not invariant within this taxon. Synthetic RVAL-(L^1^, T^6^, L^8^)-bradykinin was found to be an antagonist of BK-induced rat tail artery smooth muscle relaxation acting via the B_2_-receptor.

## 1. Introduction

Bradykinins (BKs) and related peptides (BRPs) are among the most abundant and structurally-diverse group of pharmacologically-active peptides present in anuran amphibian defensive skin secretions [1,2]. Following from the initial discovery of canonical mammalian bradykinin (BK) in the skin of the European brown frog, *Rana temporaria*, many site-substituted, truncated and/or *N*- and *C*-terminally-extended peptides have been isolated from the skins/skin secretions of representative species from the families, Ranidae, Hylidae, Bombinatoridae and Leiopelmatidae, with ranid frogs having the most diverse range of BRPs [3,4,5,6,7,8,9]. It has become increasingly clear, following the application of skin peptide biosynthetic precursor-encoding cDNA cloning techniques to this field of research, that there is a high degree of variability in mature BRP primary structures, propeptide convertase processing sites and copy numbers of BRP-encoding domains and their locations, within such precursor proteins. In fact, such is the magnitude of this variability that even between con-generic species, it is often not possible to predict any of these features with any degree of accuracy. The reasons for the high degree of primary structural variability in mature amphibian skin BRPs were unknown until recently, when it emerged, following a series of systematic studies on sub-mammalian vertebrate plasma kinins, that this heterogeneous array of amphibian BRPs appears to be modelled on those present within various taxa of their vertebrate predators, ranging from bony fishes, through several reptilian taxa, to birds and mammals [10]. Amphibians appear not to possess a plasma BRP for reasons that are unknown at present [1,2,10]. In this study, a novel *N*-terminally-extended and site-substituted BK has been found in the skin secretion of the Fujian large-headed frog (*Limnonectes fujianensis*). This BRP, named RVAL-(L^1^, T^6^, L^8^)-bradykinin, was found to be an antagonist of BK-induced relaxation of rat tail arterial smooth muscle by blocking its interaction with B_2_-receptors. The Leu (L) residue at the penultimate position was probably responsible for this effect, as has been previously reported. However, bioinformatic analysis of the *C*-terminal nonapeptide sequence (-LPPGFTPLR) of this novel peptide produced several results of general interest to molecular evolutionary biologists, which will be discussed.

## 2. Results

### 2.1. “Shotgun” Cloning of a cDNA Encoding the Biosynthetic Precursor of a Novel BRP

**Figure 1:**
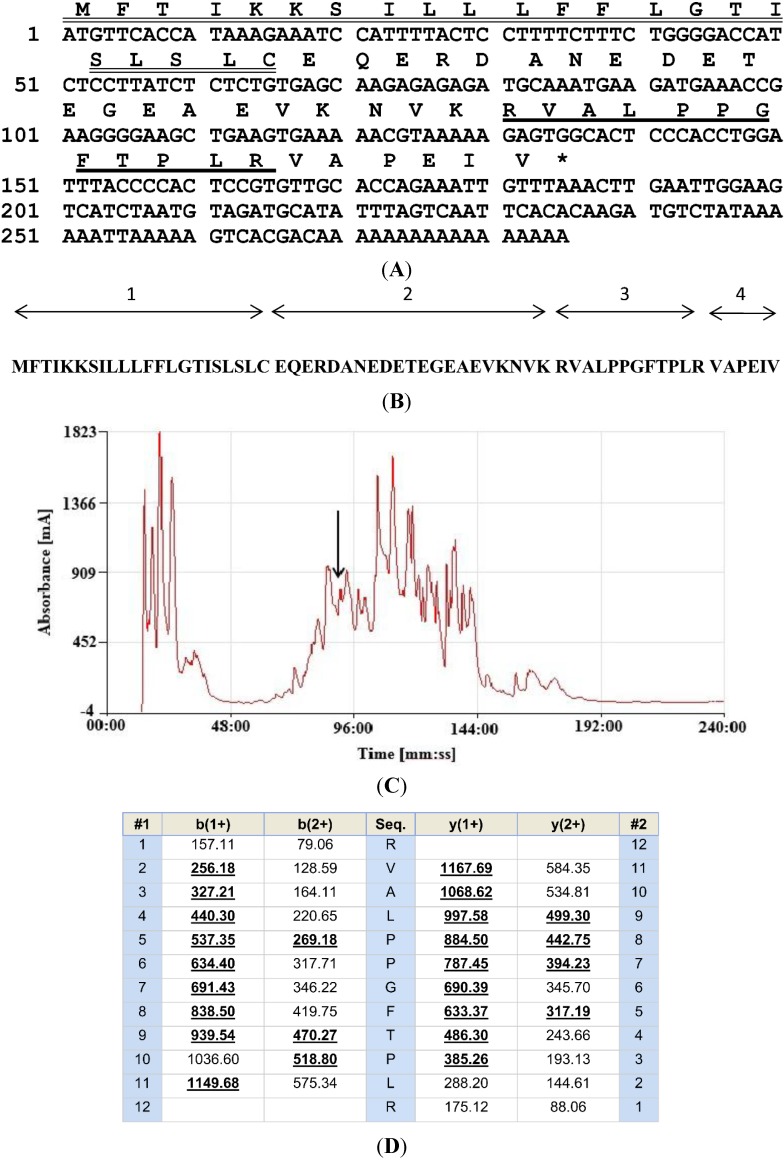
(**A**) Nucleotide and translated open-reading frame amino acid sequence of the cDNA encoding the biosynthetic precursor of the novel BRP-RVALPPGFTPLR, from *Limnonectes fujianensis* skin secretions. The putative signal peptide is double-underlined, and the mature peptide is single-underlined. The stop codon is indicated with an asterisk; (**B**) Domain architecture of the novel BRP-encoding biosynthetic precursor: (1) putative signal peptide; (2) spacer peptide; (3) mature BRP; (4) *C*-terminal extension peptide; (**C**) Reverse phase HPLC chromatogram of *Limnonectes fujianensis* skin secretions indicating the retention time/elution position of the absorbance peak corresponding to the novel BRP (arrow). The *Y*-axis represents the relative absorbance at λ 214 nm and the *X*-axis represents the retention time in minutes; (**D**) Predicted *b-* and *y*-ion series (singly- and doubly-charged) resulting from MS/MS fragmentation of the doubly-charged ion (*m*/*z* 663.24) of the novel BRP. Ions observed in MS/MS fragmentation spectra are indicated in bold typeface and are underlined. The nucleotide sequence of RVAL-(L^1^, T^6^, L^8^)-bradykinin, from the skin secretion of *Limnonectes fujianensis*, has been deposited in the EMBL Nucleotide Sequence Database under Accession Code HG 970097.

A single cDNA, whose translated open-reading frame encoded the biosynthetic precursor of a novel BRP, was consistently cloned (at least 10 times) from the skin secretion-derived cDNA library of *L. fujianensis*. The nucleotide and translated open reading frame amino acid sequences of this clone are shown in Figure 1A. The architecture of the precursor can be divided into four domains: (1) a putative signal peptide domain containing 22 amino acids; (2) an acidic amino acid-rich spacer peptide domain; (3) a putative mature peptide domain consisting of 12 amino acid residues; and (4) a *C*-terminal extension peptide domain (Figure 1B).

### 2.2. Isolation and Structural Characterisation of the Novel BRP

The reverse phase HPLC chromatogram of the lyophilised skin secretion from *L. fujianensis* is shown in Figure 1C. A sample (1 μL) from each 1-mL fraction was subjected to MALDI-TOF MS analysis to identify which contained a peptide of molecular mass coincident with that of the putative novel BRP. This was located in Fraction 90 (arrow in Figure 1C) (*m*/*z* of 1324.41 (M + H)^+^ and *m*/*z* of 663.24 (M + 2H)^2+^) (data not shown). The primary structure of this peptide was confirmed by MS/MS fragmentation sequencing using the electrospray ion-trap mass spectrometer (Figure 1D). The primary structure of the novel BRP was thus unequivocally established from a combination of molecular cloning and mass spectrometric data as RVALPPGFTPLR, and this peptide was thus named systematically as RVAL-(L^1^, T^6^, L^8^)-BK.

### 2.3. Bioinformatic Analyses of the Novel BRP, RVAL-(L^1^, T^6^, L^8^)-BK

**Figure 2:**
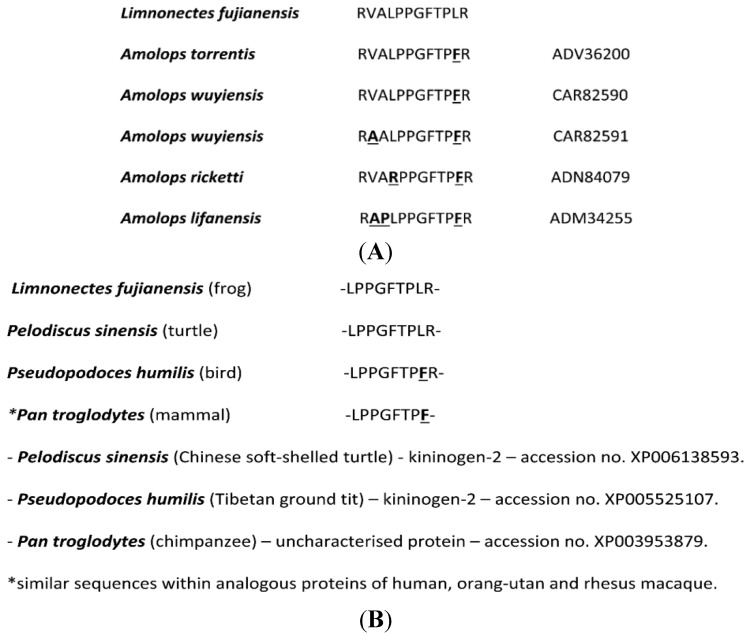
(**A**) NCBI BLAST analysis of the primary structure of the novel BRP showing the top five hits obtained. All are dodecapeptides from ranid frogs of the genus, *Amolops*. Sites of amino acid differences are in bold typeface and are underlined with database accession numbers indicated; (**B**) NCBI BLAST analysis results using the *C*-terminal nonapeptide of the novel BRP as the query. Note the identity with the BRP predicted from kininogen-2 of the Chinese soft-shelled turtle (*Pelodiscus sinensis*) and a single amino acid difference (F/L) with the homologue from kininogen-2 of the Tibetan ground tit (*Pseudopodoces humilis*). Of interest is the similar domain present within the same uncharacterised protein of primates.

The results of bioinformatic analyses using the structure of the novel BRP as a query are shown in Figure 2. The novel BRP from *L. fujianensis* skin secretion exhibited a high degree of primary structural identity with BRPs from the skins of oriental torrent frogs of the genus, *Amolops* (Figure 2A). This high degree of identity, however, did not extend to the penultimate residue (Leu (L) in *L. fujianensis* BRP and Phe (F) in all *Amolops* BRPs), which is important in BK receptor interactions [9]. The *C*-terminal nonapeptide bioactive core sequence of the novel BRP was found to be identical to the predicted BK domain within kininogen-2 of the Chinese soft-shelled turtle (*Pelodiscus sinensis*) and, with the exception of the penultimate Leu (L) residue, with a homologous domain within kininogen-2 of the Tibetan ground tit (*Pseudopodoces humilis*)—a passerine bird. A homologous domain was also observed within a conserved, but as yet uncharacterised, protein, present within monkeys and great apes, including humans (Figure 2B).

### 2.4. Rat Arterial Smooth Muscle Pharmacology of Synthetic RVAL-(L^1^, T^6^, L^8^)-BK 

**Figure 3:**
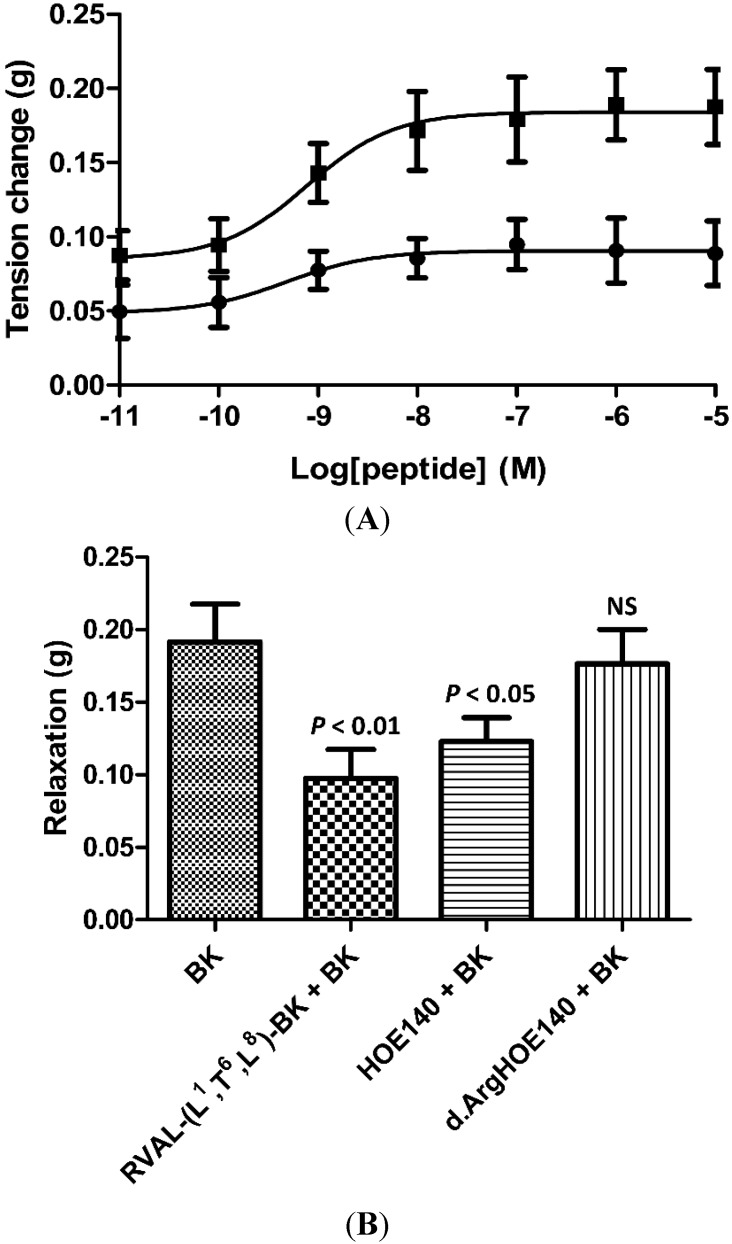
(**A**) BK dose-response curves using rat arterial smooth muscle in the absence (■) and presence (●) of the novel BRP at a single dose of 10^−6^ M; (**B**) Relaxation effect of BK on rat arterial smooth muscle at a single dose of 10^−6^ M and the effect of pre-treatment with the novel BRP (RVAL-(L^1^, T^6^, L^8^)-BK) at 10^−6^ M (*p* < 0.01), the selective BK B_2_-receptor antagonist, HOE140, at 3 × 10^−7^ M (*p* < 0.05) and the selective BK B_1_-antagonist, desArg-HOE-140, at 3 × 10^−7^ M (NS, not significant). All data points represent the mean ± SEM of six replicates.

Parallel dose-response curves (10^−11^ to 10^−5^ M), using either synthetic BK or RVAL-(L^1^, T^6^, L^8^)-BK, on rat tail arterial smooth muscle preparations, resulted in a typical dose-dependent relaxation with BK (EC_50_ 9.3 × 10^−9^ M) and no observable effect with RVAL-(L^1^, T^6^, L^8^)-BK (data not shown). In a second series of experiments, RVAL-(L^1^, T^6^, L^8^)-BK was pre-incubated with phenylephrine pre-constricted rat tail artery smooth muscle preparations at a single dose (10^−6^ M), prior to construction of dose-response curves with BK. RVAL-(L^1^, T^6^, L^8^)-BK caused significant reductions in BK-induced vasorelaxation at all BK doses tested (*p* < 0.05 at BK concentrations of 10^−11^ and 10^−10^ M; *p* < 0.01 at BK concentrations between 10^−9^ M and 10^−5^ M) (Figure 3A).

In Figure 3B, the effects of a single dose of BK (10^−6^ M) on phenylephrine pre-constricted rat tail artery smooth muscle preparations following the addition of buffer alone or with either RVAL-(L^1^, T^6^, L^8^)-BK (10^−6^ M), the specific BK B_2_-receptor antagonist, HOE 140, or the specific BK B_1_-receptor antagonist, desArg-HOE 140, both at single doses of 3 × 10^−7^ M, are shown. These data indicated that the target BK receptor for the inhibitory actions of RVAL-(L^1^, T^6^, L^8^)-BK was likely to be of the B_2_-subtype. The B_2_-receptor antagonist, HOE 140, caused a significant reduction (*p* < 0.05) in the vasorelaxant effects of BK in these preparations, with the B_1_-receptor antagonist, desArg-HOE 140, producing no significant effects. RVAL-(L^1^, T^6^, L^8^)-BK produced the most significant observed reduction in bradykinin-induced vasorelaxation (*p* < 0.01).

## 3. Discussion

Bradykinin (BK) is a peptide with one of the largest spectra of biological actions that include fundamental roles in the establishment and maintenance of inflammation, pain transmission and smooth muscle modulation, and BK and/or BRPs are widely-distributed in the tissues of all vertebrate groups so far studied—fishes, amphibians, reptiles, birds and mammals [1,2,11,12]. However, in the amphibians, unlike all other vertebrate taxa, BK and bradykinin-related peptides (BRPs) appear to be present only in defensive skin secretions and are not apparently encoded within the structure of a higher molecular weight plasma kininogen [1,2,10]. These plasma kininogens in the majority of vertebrate taxa are produced predominantly by the liver and are secreted into the circulation, where the encoded BRPs are generated on demand by plasma or tissue proteases—the kallikreins [10,12]. In contrast, the BK and BRPs of amphibian skin secretions are synthesised within the granular gland cells, and their biosynthetic precursors possess the typical organisation observed for other skin secretion peptides with the active BRP being post-translationally processed and stored in this form prior to secretion [1,2,10]. This highly-conserved precursor organisation, reflected in a similar high degree of primary structural and nucleotide sequence conservation within signal peptides of diverse species, provides the basis of primer design for use in “shotgun” cloning of skin secretion-derived cDNA libraries, even when the primary structures of the various encoded bioactive peptides are not known [13,14]. This was the strategy initially employed in the present study using such a skin secretion-derived cDNA library from the Fujian large-headed frog, *Limnonectes fujianensis*. A cDNA encoding the biosynthetic precursor of a novel BRP was repeatedly cloned, and the deduced primary structure of this peptide was confirmed by the application of mass spectrometric techniques to samples from reverse phase HPLC fractionation of skin secretions. Bioinformatic analysis of the confirmed structure using NCBI-BLAST generated interesting data. Firstly, the novel BRP was found to exhibit high degrees of structural similarity to a series of dodeca-BRPs isolated from the skin secretions of ranid frogs from the genus, *Amolops*. However, all of these possessed a Phe (F) residue in the penultimate position rather than the Leu (L) residue of the novel peptide. This is not a trivial difference in BRPs, as this positional difference can change the peptide ligand from a BK receptor agonist (F) to a receptor antagonist (L), such as has been observed for BK and its avian homologue, ornithokinin [15]. However, while this appears to be true for nonapeptide BRPs, it is an effect that apparently does not hold true for this group of *N*-terminally extended dodeca-BRPs found in *Amolops*, as these have been shown to be potent BK receptor antagonists [9]. Therefore, their *N*-terminal extensions must be playing a fundamental role in the antagonism of mammalian BK receptors. The novel dodeca-BRP described here from *L. fujianensis* behaves as an antagonist of the relaxation effects of BK in rat tail arterial smooth muscle and does so through the B_2_-receptor; an action that apparently does not require a Leu residue in the penultimate position. Secondly, the *C*-terminal nonapeptide sequence of the novel dodeca-BRP, -LPPGFTPLR, was found to be identical to the endogenous BRP encoded by kininogen-2 of the Chinese soft-shelled turtle, *Pelodiscus sinensis*, a species that is sympatric with *L. fujianensis* and which is a carnivore that feeds on frogs. This amphibian skin BRP may thus represent another example of predator taxon-specific defence. Perhaps the most interesting discovery was that this domain of the novel frog skin BRP exhibited a high degree of similarity to the BRP encoded by kininogen-2 of a passerine bird—the Tibetan ground tit (*Pseudopodoces humilis*). The avian order, Passeriformes, contains more species than all other 27 orders combined [16,17]. The only difference in amino acid sequence between this passerine bird BRP and that of the novel frog skin BRP *C*-terminal nonapeptide was the presence of a Phe rather than a Leu residue in the penultimate position. This intriguing observation, rather than leading to a dubious speculation that, in some way, the frog skin BRP is a defence against passerine bird predation, suggests that the name, ornithokinin (avian bradykinin), given originally to the chicken (*Gallus gallus*) BRP (RPPGFTPLR), is not universally applicable across this taxon. In previous reports relating to the pancreatic polypeptide (PP) hormone [18,19], it was likewise found that the homologue present in passerine bird (crow) pancreas [20] significantly-differed from the archetypal avian PP originally isolated from chicken pancreas by the presence of an *N*-terminal Ala (A) residue and a Pro (P) residue at Position 34, rather than the Gly (G) residue at Position 1 and the His (H) residue at Position 34 found in chicken, goose (*Anser anser*; order Anseriformes), ostrich (*Struthio camelus*; Struthioniformes) and herring gull (*Larus argentatus*; order Charadriiformes) peptides [21,22,23,24]. It may be that the bioinformatic analysis of this frog skin BRP has revealed that in birds, there are at least two forms of BRP, similar to the findings of previous studies on their endogenous pancreatic polypeptides. Further bioinformatic analyses of kininogen sequences archived in the NCBI database (March, 2014) with this in mind and focusing on their bradykinin-encoding domains revealed the presence of a third variant BRP in birds—(Thr^6^)-BK—once considered the archetypal BRP in crocodilians and testudines [2]. This BRP was predicted from kininogen sequences from passeriform birds—the collared flycatcher (*Ficedula albicollis*), the white-throated sparrow (*Zonotrichia albicollis*), the zebra finch (*Taeniopygia guttatus*) and the medium ground finch (*Geospiza fortis*); accipitriform birds—the saker falcon (*Falco cherrug*) and the peregrine falcon (*Falco peregrinus*); an anseriform bird—the mallard (*Anas platyrhynchos*); a columbiform bird—the rock dove (*Columba livia*); and a psittaciform bird—the budgerigar (*Melopsittacus undulatus*). Thus, these data may have implications on the evolutionary origins of birds and may suggest that this group of vertebrates is not monophyletic.

## 4. Experimental Section

### 4.1. Acquisition of Frogs and Harvesting of Skin Secretions

Specimens of the Fujian large-headed frog, *L. fujianensis* (*n* = 3, snout-to-vent length 4–7 cm), were captured during expeditions in Wuyi City of the People’s Republic of China. All frogs were adults of undetermined sex, and secretion harvesting was performed in the field, after which, the frogs were released. Skin secretion was obtained by gentle transdermal electrical stimulation of the dorsal skin, as described by Tyler *et al*., 1992 [25]. Stimulated secretions were washed from the skin using deionised water and were maintained at 4 °C prior to being snap-frozen in liquid nitrogen, lyophilised and stored at −20 °C prior to analyses.

### 4.2. “Shotgun” Cloning of a L. Fujianensis Skin Secretion-Derived cDNA Library

Five milligrams of lyophilised *L. fujianensis* skin secretion were dissolved in 1 mL of lysis/binding buffer (Dynal, Merseyside, UK) to stabilise endogenous mRNA. Polyadenylated mRNA was then trapped and isolated by use of magnetic oligo-dT Dynabeads following the protocol described by the manufacturer (Dynal, Merseyside, UK). The trapped polyadenylated mRNA template was then reverse-transcribed to generate a cDNA library, and a sample of this was subjected to 5'- and 3'-rapid amplification of cDNA end (RACE) procedures to obtain full-length bradykinin-related peptide (BRP) precursor-encoding nucleic acid sequence data using a SMART-RACE kit (Clontech, Oxford, UK) following the manufacturer’s instructions. Briefly, the 3'-RACE reactions employed a nested universal primer (supplied with the kit) and a degenerate sense primer (S1; 5'-CCCRAAKATGTTSACCTYRAAGAAA-3') (*R* = *A*/*G*; *K* = *T*/*G*; *S* = *C*/*G*; *Y* = *C*/*T*) that was designed to a highly-conserved domain within the 5'-untranslated region of previously-characterised BRP precursor encoding cDNAs from closely-related *Rana* species [8,9,26]. The 5'-RACE reactions were purified and cloned using a pGEM-T vector system (Promega Corporation, Southampton, UK) and sequenced using an ABI 3730 automated sequencer (Applied Biosystems, Foster City, CA, USA).

### 4.3. Identification and Structural Analysis of the Novel BRP Deduced from Translation of the Open-Reading Frame of Cloned cDNA 

A further 5-mg sample of lyophilised *Limnonectes fujianensis* skin secretion was dissolved in 0.5 mL of trifluoroacetic acid (TFA)/water (0.05:99.95, *v*/*v*) and clarified of microparticulates by centrifugation (2500× *g* for 5 min). The clear supernatant was carefully decanted and pumped directly onto an analytical reverse phase HPLC column (Phenomenex C-18, 25 cm × 0.45 cm), and peptides were eluted using a gradient formed from 0.05/99.95 (*v*/*v*) TFA/water to 0.05/19.95/80.00 (*v*/*v*/*v*) TFA/water/acetonitrile in 240 min at a flow rate of 1 mL/min. A Cecil CE4200 Adept (Cambridge, UK) gradient reverse phase HPLC system was employed, and fractions were collected automatically at 1-min intervals. The computed molecular mass of the novel BRP predicted from cloned cDNA was used to interrogate a mass spectral library of skin secretion peptides derived from sequential analysis of each reverse phase HPLC fraction using matrix-assisted laser desorption/ionisation, time-of-flight mass spectrometry (MALDI-TOF MS) on a linear time-of-flight Voyager DE mass spectrometer (Perseptive Biosystems, Framingham, MA, USA) in positive detection mode using α-cyano-4-hydroxycinnamic acid as the matrix. Internal mass calibration of the instrument was achieved using standard peptides of established molecular mass providing a determined accuracy of ±0.1%. The peptide with a molecular mass coincident with that of the putative novel BRP was subjected to primary structural analysis using MS/MS fragmentation sequencing on an LCQ-Fleet electrospray ion-trap mass spectrometer (Thermo Fisher Scientific, San Francisco, CA, USA).

### 4.4. Solid-Phase Peptide Synthesis of the Novel BRP

Following establishment of the unequivocal primary structure of the novel BRP, a synthetic replicate was produced by using solid phase Fmoc chemistry on a PS3 automated solid-phase synthesiser (Protein Technologies, Inc., Tucson, AZ, USA). Following cleavage from the resin and deprotection, the synthetic peptide was analysed by reverse-phase HPLC and electrospray mass spectrometry to establish both the degree of purity and the authenticity of the structure.

### 4.5. Pharmacological Evaluation of the Synthetic Novel BRP Using Rat Arterial Smooth Muscle

Male adult Wistar rats, weighing 200–250 g, were killed by CO_2_ asphyxiation, followed by cervical dislocation, in accordance with institutional animal experimentation ethics and under appropriate UK animal experimentation licences. The animals were laid on their dorsal surfaces, and the tail skin was carefully removed. The tail artery vascular bed was identified and moistened with Krebs’ solution (NaCl 118 mM, KCl 4.7 mM, NaHCO_3_ 25 mM, NaH_2_PO_4_ 1.15 mM, CaCl_2_ 2.5 mM, MgCl_2_ 1.1 mM, glucose 5.6 mM). The membrane and the connective tissue beneath the main artery were carefully removed. The proximal region of the tail artery was excised and immediately placed in ice-cold Kreb’s solution. Two mm-wide rings of artery were cut and mounted on a transducer prior to placing in 2-mL capacity organ baths containing Kreb’s solution flowing at 2 mL/min and maintained at 37 °C with constant bubbling of carbogen gas mixture (95% O_2_/5% CO_2_). Muscle rings were equilibrated for 1 h before experimental procedures were initiated. A stock of the synthetic peptide was prepared in Kreb’s solution at a concentration of 10^−4^ M. Subsequently, a range of concentrations of the test peptide (from 10^−5^ M to 10^−11^ M) was prepared by ten-fold dilutions in Kreb’s solution, to facilitate the construction of a dose-response curve. A series of the same concentrations of synthetic mammalian BK were prepared in the same manner. Peptide solutions were added separately to the rat tail arterial smooth muscle rings in the organ-baths, in increasing concentrations, with 5-min washes and 5-min equilibration periods between each dose. Dose-response curves were constructed for each peptide on individual arterial smooth muscle preparations (*n* = 6). Changes in tension of the arterial smooth muscle preparations were detected by pressure transducers connected to a PowerLab System (AD Instruments Pty Ltd., Bella Vista, Australia). Data were analysed to obtain the mean and standard error of responses by Student’s *t*-test, and dose-response curves were constructed using a best-fit algorithm through the data analysis package provided.

Two additional series of experiments were performed to further analyse the smooth muscle pharmacology of the novel BRP. (1) The inhibitory effect of the novel BRP on the dose-dependent relaxation of the arterial smooth muscle preparations induced by bradykinin in the concentration range of 10^−11^–10^−5^ M was assessed at a single pre-treatment concentration of 10^−6^ M; (2) The effects of the pre-treatment of arterial smooth muscle preparations with the specific bradykinin B_1_ receptor antagonist (desArg HOE 140) or the bradykinin B_2_ receptor antagonist (HOE 140) (Sigma-Aldrich, Dorset, UK) were assessed to compare with those observed for both the synthetic novel BRP and mammalian BK. Briefly, in the first series of experiments, the stabilised preparations were exposed to bradykinin in the concentration range of 10^−11^–10^−5^ M, with and without pre-treatment with the novel BRP at a single dose of 1 × 10^−6^ M. For these experiments, the novel BRP was added to the organ bath once a stable plateau of phenylephrine-induced constriction had been obtained, and after a further period of 10 min, bradykinin dose-response curves (10^−11^–10^−5^ M) were constructed. In a second series of experiments, the specific bradykinin receptor antagonists were employed likewise at a single dose (3 × 10^−7^ M) prior to the application of BK at a single maximally-effective dose (10^−6^ M). 

## 5. Conclusions

The novel amphibian BRP described here was chemically-synthesised and subjected to pharmacological assay using rat tail arterial smooth muscle preparations. These experiments found that the peptide effectively antagonised the smooth muscle relaxation effects induced by BK in this preparation, an effect apparently mediated through the B_2_-receptor subtype. Although the Leu residue in the penultimate position of this BRP could be implicated in this antagonism of mammalian B_2_-receptors, in a manner reflecting that demonstrated previously with ornithokinin and other Leu-containing BRPs, it is also important to note the obvious contribution of the *N*-terminal extension, as homologues from frogs of the genus, *Amolops*, that possess this feature, but have a Phe residue in the penultimate position, which are also potent antagonists of mammalian BK receptors [9,27]. 

Thus, the structure/activity requirements of ligands for mammalian BK receptors, specifically with respect to what features lead to agonism/antagonism, are more complex than previously thought, as revealed by interrogation with the plethora of unique BRPs found in amphibian skin defensive secretions. A systematic pharmacological study with such unique ligands using cell lines transfected with specific BK receptor sub-types may provide new insights into their structure/activity relationships that could have implications for future drug design. 
